# Early Post-stroke Depression and Mortality: Meta-Analysis and Meta-Regression

**DOI:** 10.3389/fpsyt.2018.00530

**Published:** 2018-11-01

**Authors:** Francesco Bartoli, Carmen Di Brita, Cristina Crocamo, Massimo Clerici, Giuseppe Carrà

**Affiliations:** ^1^Department of Medicine and Surgery, University of Milano-Bicocca, Milan, Italy; ^2^Division of Psychiatry, University College London, London, United Kingdom

**Keywords:** stroke, depression, mortality, meta-analysis, meta-regression

## Abstract

**Background:** Post-stroke depression (PSD) is a common and serious complication after stroke. In this systematic review and meta-analysis, we evaluated the association between early PSD and mortality, considering depressive symptoms occurring within the first 3 months after the neurological event.

**Methods:** This meta-analysis was conducted following Meta-analysis Of Observational Studies in Epidemiology (MOOSE) guidelines and based on studies indexed till May 2018 in PubMed and Web of Science databases. The relative risk (RR) for mortality in individuals with PSD, as compared with non-depressed ones, was estimated. Findings were pooled according to a random-effects model. Meta-regression and subgroup analyses were carried out.

**Results:** We included seven studies, accounting for 119,075 individuals, of whom 17,609 suffering from an early PSD. We found higher rates of mortality in subjects with PSD as compared with non-depressed ones (RR = 1.50; 95%CI: 1.28 to 1.75; *p* < 0.001). Heterogeneity across studies was moderate (*I*^2^ = 50.7%). Subgroup analysis showed a slightly higher effect of PSD on short-term mortality (RR = 1.70; *p* < 0.001), as compared with long-term one (RR = 1.35; *p* = 0.01). According to relevant meta-regression analyses, the estimate was influenced by sample proportion of men (*p* = 0.043).

**Conclusions:** Despite some limitations, our study shows the negative impact of early PSD on survival rates. Mechanisms underlying this association still need to be elucidated and several interpretations can be hypothesized. Future research should test if an early management of depression may increase life expectancy after stroke.

## Introduction

Stroke is the second leading cause of death worldwide, accounting for 6.3 million deaths in 2015 (1), and a major public health issue associated with an increasing global burden of disease (2) and different physical (3) and neuropsychological consequences (4). Post-stroke depression (PSD) is a common and serious complication after stroke (5), occurring in about one third of subjects, especially during the first stage after the acute event (6). The pathophysiology of PSD is probably multifactorial, deriving from a complex interaction between ischemia-induced neurobiological dysfunctions and psychological distress (7, 8). Although an early detection of PSD is a key issue in clinical practice, its identification remains challenging (9). This may be due to some neurological symptoms which could conceal primary mood abnormalities (5), as well as the lack of satisfactory diagnostic tools for case-finding (10). However, PSD represents one of the main factors limiting recovery and rehabilitation (9), predisposing to severe disability, functional and cognitive impairment, greater dependency in daily activities, and low treatment adherence (11, 12). In addition, several studies identified PSD as a potential risk factor for mortality (13–15). Consistently, a previous systematic review and meta-analysis (16), including 13 studies and 59,598 subjects, estimated a small, though significant, effect of PSD on mortality. However, findings were probably influenced by the methodological heterogeneity across studies. In particular, along with follow-up duration, high time variability for PSD assessment in different studies should be taken into account, considering that PSD frequency may change over time (5). Indeed, some studies evaluated early PSD (14), whereas other assessed depression up to several years after stroke (15). Since symptoms usually occur within the first 3 months after stroke (17), it seems important to evaluate the impact on survival of early PSD, including findings of recent studies published in this field (11, 18). We thus conducted a systematic review and meta-analysis, to investigate the association between early PSD and mortality, considering different factors that might potentially influence the estimated effect, including sample characteristics, assessment methods, follow-up duration, and quality.

## Methods

This systematic review and meta-analysis was conducted following the Meta-analysis of Observational Studies in Epidemiology (MOOSE) guidelines (19).

### Search strategy

We updated the strategy of our previous systematic review and meta-analysis (16), searching for articles indexed in PubMed and Web of Science electronic databases up to May 30th, 2018. No language restriction was set. We used the following terms for the PubMed search: “(depression [Mesh] or depression [all fields]) and (Stroke [Mesh] or post-stroke [title/abstract] or post stroke [title/abstract]) and (Mortality [Mesh] or mortal^*^ [title/abstract] or death^*^ *[title/abstract])*.” We used a similar search phrase for Web of Science database: “(depress^*^ or mood^*^ or affective^*^) and (stroke^*^ or post-stroke or poststroke) and (mortal^*^ or death^*^).”

### Eligibility criteria

Studies which compared the survival rates in adults with and without PSD, respectively, were considered eligible. To be included, studies had to assess PSD within 3 months after an acute stroke. Both ischemic and hemorrhagic stroke were considered. We excluded articles providing continuous scores based on psychometric scales for depression, without dichotomization based on a cut-off value.

### Data extraction and quality assessment

Two authors (FB and CDB) independently completed the preliminary screening, based on titles and abstracts, in order to identify potentially eligible articles. The final inclusion decision was based on full text evaluation. We built a data extraction template, including key items for all eligible studies, i.e., year of publication, country, sample size, PSD and mortality assessment methods, follow up duration, rates of mortality in individuals with and without PSD, respectively.

In addition, we performed a quality evaluation of included studies, based on items derived from the Newcastle Ottawa Scale for non-randomized studies (20). We evaluated sample representativeness, recruitment source comparability between PSD and non-PSD groups, quality of methods to assess PSD and to retrieve information on cases of death, as well as the investigation of potential confounders.

Two authors (FB and CDB) independently extracted data and assessed quality for blind check of accuracy. Discordances were resolved by consensus with other co-authors.

### Data analysis

We conducted a meta-analysis estimating the relative risk (RR) for mortality in individuals with PSD as compared with those without. We carried out a *post-hoc* stratification of data, according to study follow-up duration. Findings were pooled according to the random-effects model and were summarized using a conventional forest plot. In addition, we carried out random-effects meta-regression analyses with Hartung-Knapp modification (21), exploring whether the estimated association between PSD and mortality might vary according to different study characteristics. These included continuous variables such as sample size, mean age, male gender proportion, and follow-up duration, as well as a categorical variable distinguishing methods to assess PSD (i.e., standardized clinical interviews, psychometric scales, or clinical records). We also carried out sensitivity analyses excluding studies with potential methodological issues based on considered quality items.

Statistical significance was set at *p* < 0.05. Consistency across studies was measured using the *I*^2^ index, with standard values of 25, 50, and 75% taken to indicate low, moderate and high levels of heterogeneity, respectively (22). Analyses were performed using the Stata statistical software package (release 14).

## Results

### Study selection

**Figure 1:**
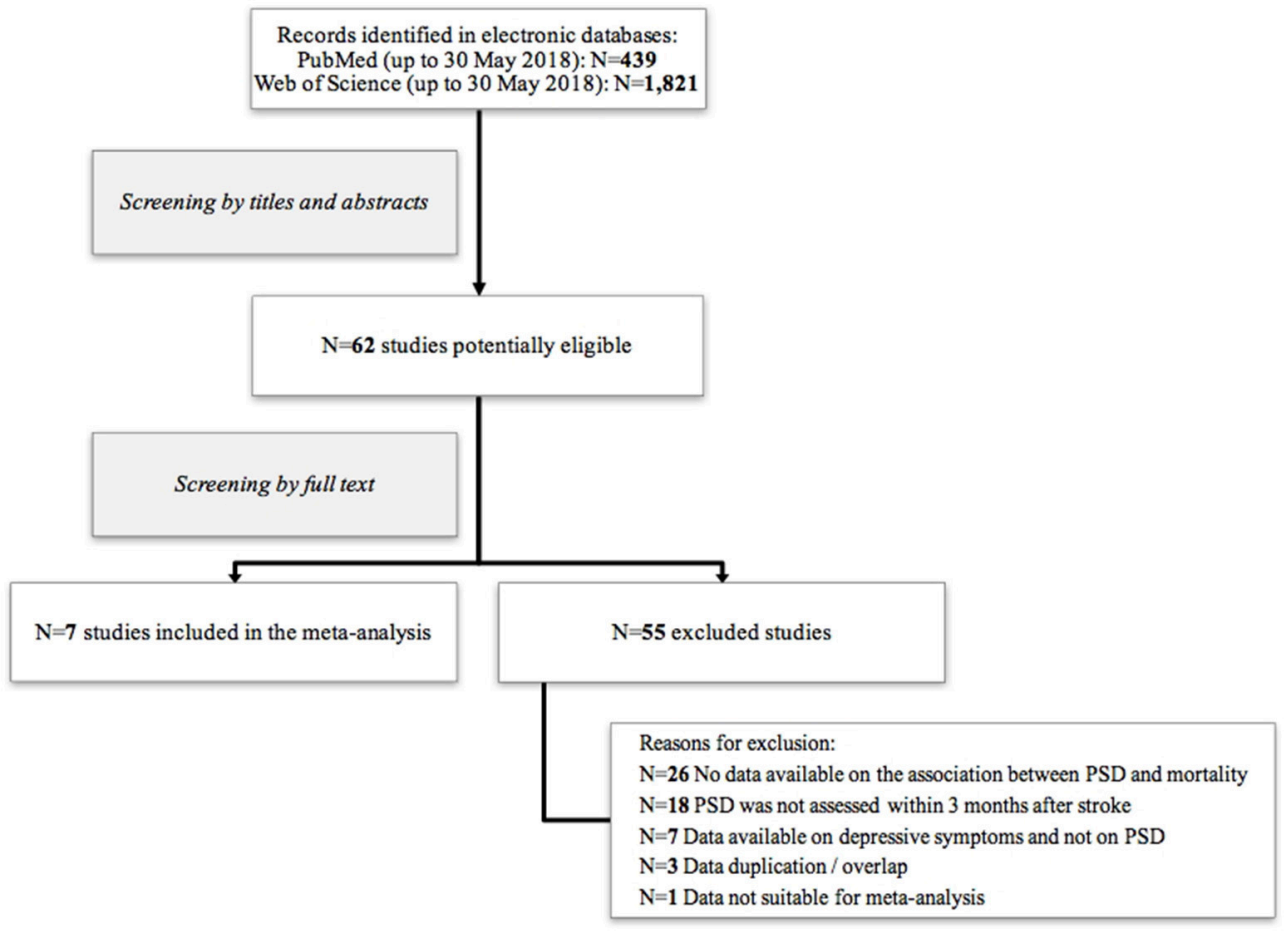
Flow chart of included/excluded studies.

Our search generated 439 and 1,821 records from PubMed and Web of Science databases, respectively. The preliminary evaluation based on titles and abstracts, identified 62 potentially eligible studies. Further screening of full texts allowed to exclude 55 articles. Seven studies met eligibility criteria and were included in our meta-analysis (11, 14, 18, 23–26). Details on study selection process are shown in Figure 1.

### Study characteristics and quality assessment

All included studies, published between 1993 (24, 25) and 2016 (18, 23), were written in English. Full characteristics are shown in Table 1. Most of studies could be considered sufficiently representative since they recruited subjects selected from specific catchment areas. However, one study (14) selected participants from a randomized-controlled trial on psychological treatments after stroke, who are likely to be different from the standard clinical population suffering from stroke. Moreover, all studies recruited both PSD and non-PSD individuals from the same setting, thus guaranteeing recruitment source comparability between cases and controls. In addition, all studies but one (23), deriving diagnosis from register-based information, tested PSD using clinical interviews or psychometric scales. One study (26) used only the first question of the Hamilton Depression Rating Scale, thus limiting its psychometric validity. In terms of mortality, adequate sources, such as death registries, were used in most of studies. In two studies (24, 25), information on mortality was unclear and probably not sufficiently valid, since the vital status of patients at follow-up could not be checked in a high proportion of cases (>10%). Finally, all studies but two (24, 25), which provided only unadjusted data, estimated the association between PSD and mortality after controlling for possible demographic and clinical confounders. Quality evaluation is summarized in Table 2.

**Table 1:** Characteristics of included studies.

**Study (reference)**	**City, Country**	**Participants**			**PSD assessment**	**Follow-up (yrs.)**
		***N***	**Mean age (yrs.)**	**Men (%)**		
Ayerbe et al. (11)	London, UK	1,354	68.1	54.0	HADS	5
de Mello et al. (18)	São Paulo, Brazil	191	63	60.2	PHQ-9	1
House et al. (14)	Leeds and Bradford, UK	448^*^	70.7	53.8	PSE	2
Jørgensen et al. (23)	Denmark	116,569^**^	68.7^**^	53.8^**^	Clinical registries	2
Morris et al. (24)	Baltimore, USA	91	60.4	59.3	PSE	10
Morris et al. (25)	New South Wales, Australia	84	71.1	53.6	CIDI	1.25
Willey et al. (26)	New York, USA	340	68.8	43.5	Single item from HRSD	5

BDI-II, Beck Depression Inventory-Second Edition; CIDI, Composite International Diagnostic Interview; HADS, Hospital Anxiety and Depression Scale; HRSD, Hamilton Rating Scale for Depression; PHQ-9, Patient Health Questionnaire; PSE, Present State Examination.

* Data from 446 individuals were included in the meta-analysis, since data from two subjects were missing.

** *Data from the subsample of subjects survived and evaluated for depression within 3 months after stroke*.

**Table 2:** Quality assessment and sensitivity analyses.

**Study (reference)**	**Sample representativeness**	**Recruitment comparability**	**Depression assessment**	**Information on mortality**	**Control for confounders**
Ayerbe et al. (11)	(+)	(+)	(+)	(+)	(+)
de Mello et al. (18)	(+)	(+)	(+)	(+)	(+)
House et al. (14)	(–)	(+)	(+)	(+)	(+)
Jørgensen et al. (23)	(+)	(+)	(–)	(+)	(+)
Morris et al. (24)	(+)	(+)	(+)	(–)	(–)
Morris et al. (25)	(+)	(+)	(+)	(–)	(–)
Willey et al. (26)	(+)	(+)	(–)	(+)	(+)
RR (95%CI) Sensitivity analyses	1.49 (1.25 to 1.78)	–	1.62 (1.27 to 2.05)	1.45 (1.21 to 1.74)	1.45 (1.21 to 1.74)

### Synthesis of results

**Figure 2:**
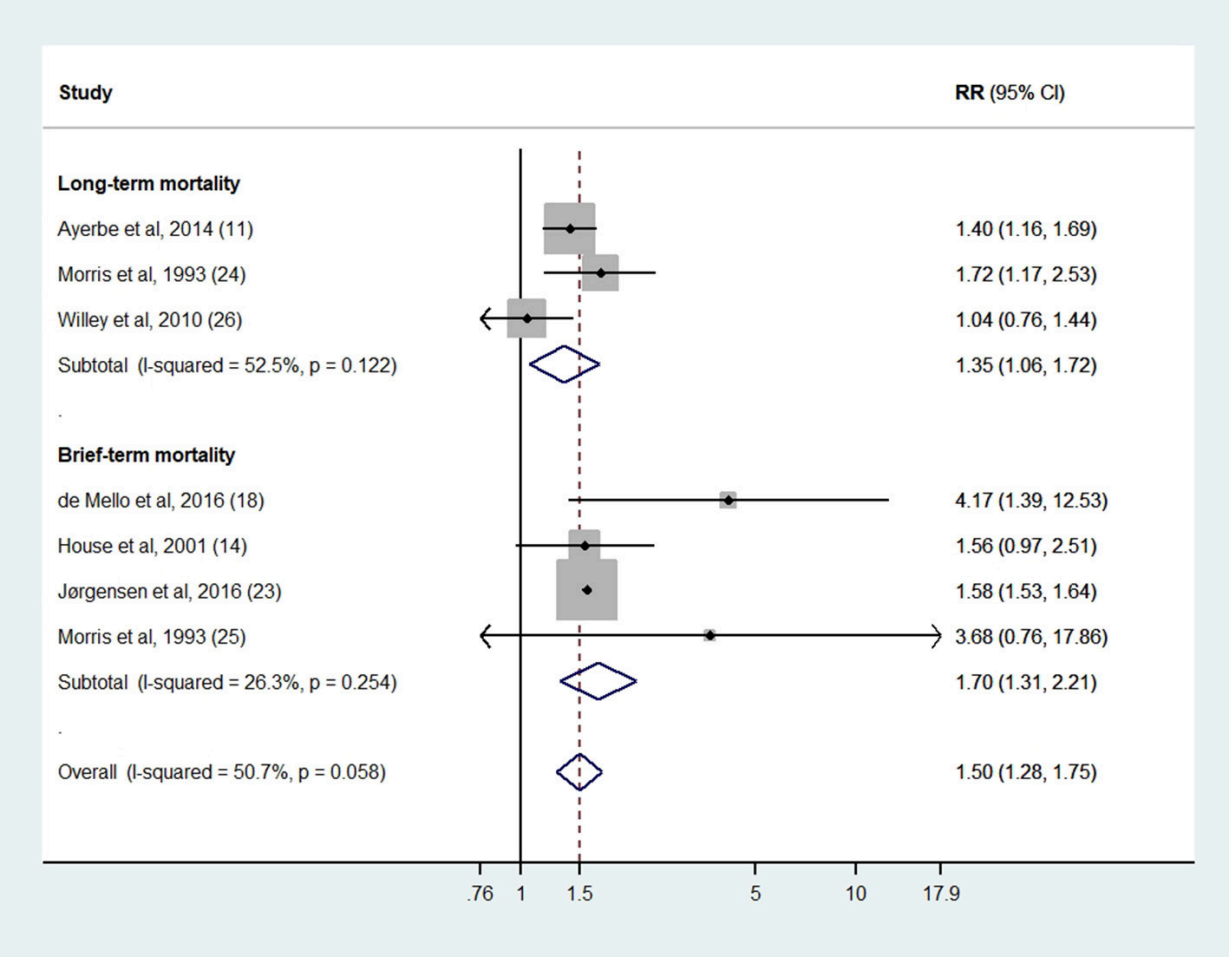
Association between early post-stroke depression and mortality. Short-term mortality = 1 or 2 years after stroke; Long-term mortality = 5 or more years after stroke.

Seven studies (11, 14, 18, 23–26) based on 119,075 individuals (17,609 suffering from PSD and 101,466 non-depressed individuals) had data suitable to estimate the random-effect RR. We used unpublished information provided by the authors from four studies (11, 14, 23, 26). As a whole, 3,588 cases of death at follow-up were detected from PSD samples and 12,906 from comparison groups. Random-effects meta-analysis estimated higher rates of mortality in subjects suffering from PSD, as compared with non-depressed ones (RR = 1.50; 95%CI: 1.28 to 1.75; *p* < 0.001), with moderate heterogeneity across studies (*I*^2^ = 50.7%) (Figure 2). The subgroup analysis showed a slightly higher effect size in studies testing mortality within 2 years after stroke (RR = 1.70; *p* < 0.001), as compared with those based on a longer period of observation (RR = 1.35; *p* = 0.01) (Figure 2). However, a relevant meta-regression analysis showed no influence of follow-up duration on the estimated effect size (ß = −0.016; *p* = 0.711). Other meta-regression analyses, considering sample size, mean age, and PSD assessment methods, did not show any significant influence on effect size, apart from the proportion of recruited men (ß = 0.039; *p* = 0.043). In addition, sensitivity analyses based on quality issues confirmed the significant relationship between early PSD and lower life expectancy, with relative risk of mortality varying between 1.45 and 1.62 (Table 2).

## Discussion

### Summary and interpretation of findings

The current systematic review and meta-analysis, based on seven studies (11, 14, 18, 23–26), highlights the impact of early depression after stroke on survival rates. To our knowledge, this is the first meta-analysis estimating a higher risk of mortality in people suffering from PSD, considering depressive symptoms occurring in the early stage after the acute event. Subjects with early PSD had a risk of death about 1.5 higher as compared with non-depressed individuals, considering both short- and long-term mortality. Nevertheless, a comprehensive interpretation of these findings should take into account quality issues of included studies and a partial inconsistency of findings. Considering both internal and external validity (27, 28), we found that some studies included in this meta-analysis might have been influenced by the lack of representativeness (14) and control for confounders (24, 25), as well as by the potential low accuracy of PSD diagnosis (23) and mortality assessment at follow-up (24, 25). Nevertheless, quality-based sensitivity analyses seem consistent with the overall estimation.

In addition, our results should be interpreted considering the statistical heterogeneity, likely to be due to systematic differences in important methodological characteristics, such as target population, PSD definition, follow-up duration. The estimated excess of mortality in early PSD could not be explained by heterogeneity attributable to most of study characteristics, apart from gender. It should be considered that the strength of the relationship between PSD and mortality might be higher among men, although available data did not allow stratifying findings according to gender.

Even considering quality issues and heterogeneity, evidence from this meta-analysis is in line with findings of a previous systematic review and meta-analysis, showing an association between depression, assessed at any time after stroke, and mortality (16). Moreover, similar findings were reported in individuals with heart failure, in which depression was associated with higher risk of mortality (29). Our results are consistent with epidemiological evidence, showing the impact of depression on poor outcomes after acute vascular events (26).

Pathophysiological mechanisms which can explain the association between PSD and increased risk of mortality, remain to be clarified although several possible interpretations could be postulated. A first hypothesis may be related to depression effects on cardiovascular risk, possibly leading to recurrent cerebrovascular events and higher mortality. Indeed, individuals suffering from depressive disorders are more prone to unhealthy lifestyle behaviors, including higher rates of smoking, alcohol intake, physical inactivity, and poor dietary patterns (30, 31). Consistently, it should be considered that individuals with depression are generally less likely to be adherent and compliant to prescribed treatments and medications, being thus more vulnerable to poor clinical consequences (11, 32). Second, early depressive symptoms may occur more frequently in subjects with severe stroke-related consequences, such as disability, neurocognitive deficits, and functional impairment (33, 34). Thus, depression might be significantly related to stroke severity, that, in turn, might increase the risk of death. Third, evidence of pharmacological treatment for PSD have produced mixed results (35, 36) and benefits of selective serotonin reuptake inhibitors (SSRIs) have been questioned, considering the possible association with an even increased mortality (37). Consistently, even if antidepressants have shown to improve depressive symptoms and functional outcomes (38, 39), some studies estimated a higher likelihood of overall major bleeding and an increased risk of death in subjects treated with SSRIs (40). Thus, the excess of mortality in individuals with PSD might be partially explained by possible negative effects of a SSRI treatment on survival. Finally, mortality could be attributable also to non-natural causes of death. Stroke survivors show higher risk of death by suicide, especially if they suffer from current or lifetime mood disorder, recurrent stroke, and cognitive impairment (41). A recent systematic review and meta-analysis (42) including 10,400 stroke survivors, estimated that a clinically meaningful suicidal ideation may occur in about one out of eight individuals after the neurological event. Consistently, it has been reported that individuals suffering from stroke have rates of suicide attempts twice as high as the general population (43, 44).

### Limitations

Although the current meta-analysis provided preliminary evidence on PSD effect on mortality, caution should be used in interpreting our findings, due to some limitations.

First, we could not systematically control for treatment-related factors, such as antidepressant medication as well as other interventions for depression prevention and management (5), which might influence the association between PSD and survival rates. This seems a key issue since previous studies highlighted a possible effect of antidepressants on mortality after stroke (40).

Second, it should be considered that there is not a gold standard for PSD diagnosis and that standardized scales, combining depressive and somatic items, are not currently available (10). Consistently, we found variability across studies in terms of assessment tools for depression, varying from clinical interviews such as PSE (14, 24) and CIDI (25), to psychometric scales (11, 18, 26). Diagnostic issues arise from specific features of stroke survivors, in which neurological symptoms and neurocognitive deficits could hide symptoms of independent mood disorders (5).

Finally, even if the number of subjects and cases included in this meta-analysis is adequate, only seven studies met inclusion criteria. Since < 10 studies were included in our meta-analysis (45), we could not assess risk of publication bias by using appropriate statistical approaches (46).

### Clinical implications

Findings of this meta-analysis indirectly support the need of a regular assessment of depressive symptoms in subjects with stroke. Early depression after stroke often remains underdiagnosed and untreated, due to the lack of a gold standard for PSD assessment as well as of diagnostic issues related to clinical characteristics of this special population (10, 17). For example, stroke-related neurological symptoms such as aprosodic speech, abulia, or flat affect may hinder the clinical evaluation of depressive symptoms, whereas aphasia may lead to an underestimation of PSD (5). Consistently, evidence on pharmacological treatment of depression after stroke is limited and little is known about possible long-term adverse effects (47). In addition, further research is needed to explore efficacy of non-pharmacological interventions and prevention strategies for PSD. Various psychosocial, behavioral, and rehabilitative interventions (48–50) have been tested and may be possibly effective in reducing depressive symptoms. Although important gaps in terms of the most effective treatment for PSD remain, a greater awareness on depression after stroke and its impact on survival among patients, families, and health professionals, could facilitate recognition, earlier treatment, and improved outcomes (51).

## Conclusions

Despite some limitations, our systematic review and meta-analysis suggests the potential impact of early PSD in influencing mortality of stroke survivors. Although the underlying mechanisms of this relationship remain unknown, PSD seems to negatively influence both short- and long-term life expectancy. Further research is required to evaluate whether an early management of mood disorders in individuals with stroke may improve their outcomes and related mortality gap.

## Author contributions

All authors made substantial contributions to the intellectual content of the paper. FB and GC: conception and design; FB and CD: search and screening, data extraction, dataset management, and drafting of the manuscript; FB and CC: statistical analysis and interpretation of data; GC, MC, and CC: critical revision of the manuscript; GC and MC: supervision. All authors gave approval of the final version of the manuscript.

### Conflict of interest statement

The authors declare that the research was conducted in the absence of any commercial or financial relationships that could be construed as a potential conflict of interest.

## References

[1] GBD 2015 Mortality and Causes of Death Collaborators Global, regional, and national life expectancy, all-cause mortality, and cause-specific mortality for 249 causes of death, 1980-2015: a systematic analysis for the Global Burden of Disease Study 2015. Lancet (2016) 388:1459–544. 10.1016/S0140-6736(16)31012-1 27733281 PMC5388903

[2] Feigin VL Forouzanfar MH Krishnamurthi R Mensah GA Connor M Bennett DA. Global and regional burden of stroke during 1990-2010: findings from the Global Burden of Disease Study 2010. Lancet (2014) 383:245–54. 10.1016/S0140-6736(13)61953-4 24449944 PMC4181600

[3] Kumar S Selim MH Caplan LR. Medical complications after stroke. Lancet Neurol. (2010) 9:105–18. 10.1016/S1474-4422(09)70266-2 20083041

[4] Hackett ML Köhler S O'Brien JT Mead GE Neuropsychiatric outcomes of stroke. Lancet Neurol. (2014) 13:525–34. 10.1016/S1474-4422(14)70016-X 24685278

[5] Towfighi A Ovbiagele B El Husseini N Hackett ML Jorge RE Kissela BM. Poststroke depression: a scientific statement for healthcare professionals from the American Heart Association/American Stroke Association. Stroke. (2017) 48:e30–43. 10.1161/STR.0000000000000113 27932603

[6] Hackett ML Pickles K. Part I: frequency of depression after stroke: an updated systematic review and meta-analysis of observational studies. Int J Stroke. (2014) 9:1017–25. 10.1111/ijs.12357 25117911

[7] Robinson RG Jorge RE. Post-stroke depression: a review. Am J Psychiatry (2016) 173:221–31. 10.1176/appi.ajp.2015.15030363 26684921

[8] Villa RF Ferrari F Moretti A. Post-stroke depression: mechanisms and pharmacological treatment. Pharmacol Ther. (2018) 184:131–44. 10.1016/j.pharmthera.2017.11.005 29128343

[9] Das J Rajanikant GK. Post stroke depression: the sequelae of cerebral stroke. Neurosci Biobehav Rev. (2018) 90:104–14. 10.1016/j.neubiorev.2018.04.005 29656030

[10] Meader N Moe-Byrne T Llewellyn A Mitchell AJ Screening for post stroke major depression: a meta-analysis of diagnostic validity studies. J Neurol Neurosurg Psychiatry (2014) 85:198–206. 10.1136/jnnp-2012-304194 23385849

[11] Ayerbe L Ayis S Crichton SL Rudd AG Wolfe CD. Explanatory factors for the increased mortality of stroke patients with depression. Neurology (2014) 83:2007–12. 10.1212/WNL.0000000000001029 25355829 PMC4248453

[12] De Ryck A Brouns R Fransen E Geurden M Van Gestel G Wilssens I A prospective study on the prevalence and risk factors of post-stroke depression. Cerebrovasc Dis Extra (2013) 3:1–13. 10.1159/000345557 23626594 PMC3567876

[13] Ellis C Zhao Y Egede LE. Depression and increased risk of death in adults with stroke. J Psychosom Res. (2010) 68:545–51. 10.1016/j.jpsychores.2009.11.006 20488271 PMC2874721

[14] House A Knapp P Bamford J Vail A. Mortality at 12 and 24 months after stroke may be associated with depressive symptoms at 1 month. Stroke (2001) 32:696–701. 10.1161/01.STR.32.3.696 11239189

[15] Williams LS Ghose SS Swindle RW. Depression and other mental health diagnoses increase mortality risk after ischemic stroke. Am J Psychiatry (2004) 161:1090–5. 10.1176/appi.ajp.161.6.1090 15169698

[16] Bartoli F Lillia N Lax A Crocamo C Mantero V Carrà G. Depression after stroke and risk of mortality: a systematic review and meta-analysis. Stroke Res Treat. (2013) 2013:862978. 10.1155/2013/862978 23533964 PMC3606772

[17] Espárrago Llorca G Castilla-Guerra L Fernández Moreno MC Ruiz Doblado S Jiménez Hernández MD. Post-stroke depression: an update. Neurologia (2015) 30:23–31. 10.1016/j.nrl.2012.06.008 22901370

[18] de Mello RF Santos Ide S Alencar AP Benseñor IM Lotufo PA Goulart AC. Major depression as a predictor of poor long-term survival in a Brazilian stroke cohort (Study of Stroke Mortality and Morbidity in Adults) EMMA study. J Stroke Cerebrovasc Dis. (2016) 25:618–25. 10.1016/j.jstrokecerebrovasdis.2015.11.021 26725125

[19] Stroup DF Berlin JA Morton SC Olkin I Williamson GD Rennie D. Meta-analysis of observational studies in epidemiology: a proposal for reporting. Meta-analysis Of Observational Studies in Epidemiology (MOOSE) group. JAMA (2000) 283:2008–12. 10.1001/jama.283.15.2008 10789670

[20] Wells GA Shea B O'Connell D Peterson J Welch V Losos M The Newcastle-Ottawa Scale (NOS) for Assessing the Quality of Nonrandomized Studies in Meta-Analyses. Available online at: http://www.ohri.ca/programs/clinical_epidemiology/oxford.asp (Accessed September 07, 2018).

[21] Knapp G Hartung J. Improved tests for a random effects meta-regression with a single covariate. Stat Med. (2003) 22:2693–710. 10.1002/sim.1482 12939780

[22] Higgins JP Thompson SG Deeks JJ Altman DG. Measuring inconsistency in meta-analyses. BMJ (2003) 327:557–60. 10.1136/bmj.327.7414.557 12958120 PMC192859

[23] Jørgensen TS Wium-Andersen IK Wium-Andersen MK Jørgensen MB Prescott E Maartensson S. Incidence of depression after stroke, and associated risk factors and mortality outcomes, in a large cohort of Danish patients. JAMA Psychiatry (2016) 73:1032–40. 10.1001/jamapsychiatry.2016.1932 27603000

[24] Morris PL Robinson RG Andrzejewski P Samuels J Price TR. Association of depression with 10-year poststroke mortality. Am J Psychiatry (1993) 150:124–9. 10.1176/ajp.150.1.124 8417554

[25] Morris PL Robinson RG Samuels J. Depression, introversion and mortality following stroke. Aust N Z J Psychiatry (1993) 27:443–9. 10.3109/00048679309075801 8250788

[26] Willey JZ Disla N Moon YP Paik MC Sacco RL Boden-Albala B. Early depressed mood after stroke predicts long-term disability: the Northern Manhattan Stroke Study (NOMASS). Stroke (2010) 41:1896–900. 10.1161/STROKEAHA.110.583997 20671256 PMC2932858

[27] Egger M Schneider M Davey Smith G. Spurious precision? Meta-analysis of observational studies. BMJ (1998) 316:140–4. 10.1136/bmj.316.7125.140 9462324 PMC2665367

[28] Grimes DA Schulz KF. Bias and causal associations in observational research. Lancet (2002) 359:248–52. 10.1016/S0140-6736(02)07451-2 11812579

[29] Gathright EC Goldstein CM Josephson RA Hughes JW. Depression increases the risk of mortality in patients with heart failure: a meta-analysis. J Psychosom Res. (2017) 94:82–9. 10.1016/j.jpsychores.2017.01.010 28183407 PMC5370194

[30] Barth J Schumacher M Herrmann-Lingen C. Depression as a risk factor for mortality in patients with coronary heart disease: a meta-analysis. Psychosom Med. (2004) 66:802–13. 10.1097/01.psy.0000146332.53619.b2 15564343

[31] Hare DL Toukhsati SR Johansson P Jaarsma T. Depression and cardiovascular disease: a clinical review. Eur Heart J. (2014) 35:1365–72. 10.1093/eurheartj/eht462 24282187

[32] Grenard JL Munjas BA Adams JL Suttorp M Maglione M McGlynn EA. Depression and medication adherence in the treatment of chronic diseases in the United States: a meta-analysis. J General Inter Med. (2011) 26:1175–82. 10.1007/s11606-011-1704-y 21533823 PMC3181287

[33] Hackett ML Anderson C. Predictors of depression after stroke: a systematic review of observational studies. Stroke (2005) 36:2296–301. 10.1161/01.STR.0000183622.75135.a4 16179565

[34] Nys GM van Zandvoort MJ van der Worp HB de Haan EH de Kort PL Kappelle LJ. Early depressive symptoms after stroke: neuropsychological correlates and lesion characteristics. J Neurol Sci. (2005) 228:27–33. 10.1016/j.jns.2004.09.031 15607207

[35] Hackett ML Anderson CS House AO. Management of depression after stroke: a systematic review of pharmacological therapies. Stroke (2005) 36:1098–103. 10.1161/01.STR.0000162391.27991.9d 15802637

[36] Paolucci S. Advances in antidepressants for treating post-stroke depression. Expert Opin Pharmacother. (2017) 18:1011–7. 10.1080/14656566.2017.1334765 28535081

[37] Bartoli F Paolucci S. Association of depression and SSRIs with mortality after stroke. Neurology (2014) 83:1998–9. 10.1212/WNL.0000000000001040 25355834

[38] Jorge RE Robinson RG Arndt S Starkstein S. Mortality and poststroke depression: a placebo-controlled trial of antidepressants. Am J Psychiatry (2003) 160:1823–9. 10.1176/appi.ajp.160.10.1823 14514497

[39] Mortensen JK Johnsen SP Larsson H Andersen G. Early antidepressant treatment and all-cause 30-day mortality in patients with ischemic stroke. Cerebrovasc Dis. (2015) 40:81–90. 10.1159/000435819 26184925

[40] Mortensen JK Larsson H Johnsen SP Andersen G. Post stroke use of selective serotonin reuptake inhibitors and clinical outcome among patients with ischemic stroke: a nationwide propensity score-matched follow-up study. Stroke (2013) 44:420–6. 10.1161/STROKEAHA.112.674242 23306326

[41] Pompili M Venturini P Campi S Seretti ME Montebovi F Lamis DA. Do stroke patients have an increased risk of developing suicidal ideation or dying by suicide? An overview of the current literature. CNS Neurosci Ther. (2012) 18:711–21. 10.1111/j.1755-5949.2012.00364.x 22943140 PMC6493438

[42] Bartoli F Pompili M Lillia N Crocamo C Salemi G Clerici M. Rates and correlates of suicidal ideation among stroke survivors: a meta-analysis. J Neurol Neurosurg Psychiatry (2017) 88:498–504. 10.1136/jnnp-2017-315660 28331011

[43] Eriksson M Glader EL Norrving B Asplund K. Poststroke suicide attempts and completed suicides: a socioeconomic and nationwide perspective. Neurology (2015) 84:1732–8. 10.1212/WNL.0000000000001514 25832661 PMC4424126

[44] Harnod T Lin CL Kao CH. Risk of suicide attempt in poststroke patients: a population-based cohort study. J Am Heart Assoc. (2018) 7:e007830. 10.1161/JAHA.117.007830 29321162 PMC5850170

[45] Sterne JAC Egger M Moher D Addressing reporting biases. In: Higgins JP Green S editors. The Cochrane Handbook for Systematic Reviews of Interventions. Chichester: John Wiley & Sons, Ltd (2008). 10.1002/9780470712184.ch10

[46] Egger M Davey Smith G Schneider M Minder C. Bias in meta-analysis detected by a simple, graphical test. BMJ (1997) 315:629–34. 10.1136/bmj.315.7109.629 9310563 PMC2127453

[47] Hackett ML Anderson CS House A Xia J Interventions for treating depression after stroke. Cochrane Database Syst Rev. (2008) 4:CD003437 10.1002/14651858.CD003437.pub3 18843644

[48] Baker C Worrall L Rose M Hudson K Ryan B O'Byrne L. A systematic review of rehabilitation interventions to prevent and treat depression in post-stroke aphasia. Disabil Rehabil. (2018) 40:1870–92. 10.1080/09638288.2017.1315181 28420284

[49] Mitchell PH Veith RC Becker KJ Buzaitis A Cain KC Fruin M. Brief psychosocial-behavioral intervention with antidepressant reduces poststroke depression significantly more than usual care with antidepressant: living well with stroke: randomized, controlled trial. Stroke (2009) 40:3073–8. 10.1161/STROKEAHA.109.549808 19661478 PMC2777736

[50] Wang SB Wang YY Zhang QE Wu SL Ng CH Ungvari GS. Cognitive behavioral therapy for post-stroke depression: a meta-analysis. J Affect Disord. (2018) 235:589–96. 10.1016/j.jad.2018.04.011 29704854

[51] Anderson CS. Depression After Stroke-Frequency, Risk Factors, and Mortality Outcomes. JAMA Psychiatry (2016) 73:1013–4. 10.1001/jamapsychiatry.2016.1868 27603767

